# Assessment of Cognitive-Motor Performance Costs, Task Prioritization, and Adaptation to Dishwashing Under Increased Demand in Older Women With Arthritis

**DOI:** 10.1093/geroni/igaa059

**Published:** 2020-11-27

**Authors:** Shannon T Mejía, Karen E Nielsen, Vineet Raichur, Alicia G Carmichael, Eugene Tavares, Jennie Jarvis, Jacqui Smith, Richard Gonzalez

**Affiliations:** 1 Department of Kinesiology and Community Health, University of Illinois at Urbana-Champaign, Champaign, Illinois, US; 2 Department of Population Health Sciences, School of Public Health, Georgia State University, Atlanta, Georgia, US; 3 Institute for Social Research, University of Michigan, Ann Arbor, Michigan, US; 4 Procter & Gamble, Cincinnati, Ohio, US

**Keywords:** Arthritis, Dual task, Executive function, IADLs, Physical ability

## Abstract

**Background and Objectives:**

Hand arthritis can limit upper-limb instrumental activities of daily living (IADLs) and require the recruitment of additional cognitive and motor resources to support performance. We devised a dual-task protocol for dishwashing to examine cognitive-motor performance costs and prioritizations under increased demands, processes of adaptation, and individual differences in performance costs.

**Research Design and Methods:**

Sixty women with hand arthritis (aged 60–91) completed a standardized dishwashing protocol. Motor demand was increased via the properties of the soap dispenser. Cognitive demand was increased using audial attention and response inhibition tasks. The protocol was completed twice per lab visit on 3 occasions. Response time and dishwashing time provided measures of cognitive and motor task performance. Prioritization was determined by comparing the magnitude of dual-task cost (DTC) across tasks. Adaptation to the dishwashing protocol and novel dispenser was assessed by change in DTC across lab visits. Individual differences in cognitive and physical ability were assessed with the trail making B test and gait speed.

**Results:**

Estimates from linear mixed-effects models revealed that response time increased, whereas dishwashing time decreased, during the dual-task study stages. Cognitive-motor prioritization effects were most pronounced among women with lower cognitive and physical ability. Evidence of prioritization and individual differences in DTC diminished across lab visits.

**Discussion and Implications:**

The pattern of results suggests that older women with arthritis prioritize the motor over cognitive components of dishwashing, a common IADL. Adaptation across lab visits resulted in improved performance, reduced evidence of prioritization, and attenuated differences in DTC across physical and cognitive abilities.

**Translational Significance:** This study brings a common instrumental activity of daily living (IADL)—washing dishes by hand—into a laboratory setting to examine the prioritization of cognitive and motor resources. The sample of older women with arthritis prioritized the motor task of washing dishes over the completion of a cognitive task, suggesting that IADL support should be provided for both the cognitive and motor components of the task. This study highlights the potential to gain a richer understanding of how multiple factors simultaneously affect IADL performance.

## Background and Objectives

The onset of limitations in the ability to perform instrumental activities of daily living (IADLs) decreases the likelihood of living independently. Arthritis and detriments in cognitive and physical ability are leading drivers of the onset of IADL disability (Baker et al., 2017; Rajan et al., 2013). Osteoarthritis is the most common form of arthritis. Of affected peripheral joints, osteoarthritis in the hand is most common. The age-standardized prevalence of hand osteoarthritis in women older than age 40 is 44.2%, with 15.9% of women also being symptomatic (Haugen et al., 2011). Although performing simple activities in daily life such as housekeeping and meal preparation requires both cognitive and motor resources, little is known about the tradeoff and prioritization of these resources within the ecology of the task itself (Wesson et al., 2016). This tradeoff is especially important among individuals with arthritis, who are at higher risk of activity limitations (Theis et al., 2019). Older individuals with arthritis often select and adapt their daily routines to accommodate their limitations (Fried et al., 1996; Gignac et al., 2002; Zhang et al., 2020). In the current study, we apply an innovative upper-limb cognitive-motor dual-task protocol to examine the cognitive-motor performance costs of dishwashing, an everyday chore, in a sample of older women with diagnoses of arthritis. Specifically, we investigated costs from the increased motor and cognitive demand, the prioritization of the motor and cognitive tasks, and factors associated with individual differences in cognitive-motor dual-task performance.

The risk of limitations in the ability to complete IADLs increases with age because processes of aging drive not only declines in physical ability, strength, and balance, but also the cognitive processes essential for multitasking—such as processing speed, working memory, and response inhibition (Li et al., 2018; Rapp et al., 2006). IADL performance involves both motor processes as well as executive function—cognitive processes that involve attention, working memory, and response inhibition (Jefferson et al., 2006). IADLs also exemplify multitasking contexts in daily life (Rajan et al., 2013). Response inhibition, in particular, has been identified as the strongest predictor of IADL integrity (Nguyen et al., 2020). Meanwhile, decreased muscle mass, hand weakness, and arthritis increase the risk of IADL limitations (Lee et al., 2018).

To date, IADL performance has been limited to self-reports and lab-based assessments of single tasks. Although timed IADL performance is associated with individual differences in cognitive ability, less is known about how cognitive and motor performance of IADLs may respond to increased demand during an activity (Czaja et al., 2017; Wesson et al., 2016). Upper-limb performance, recently shown to exhibit cognitive-motor interference (Bank et al., 2018), is an essential and understudied dimension of IADLs. In this study, we examine a common upper-limb task—dishwashing—within a laboratory setting. We devised a standardized laboratory protocol that included dual-task trials to investigate cognitive-motor performance costs of increased cognitive and motor task demand.

Based on the assumption that individuals have finite resources to complete IADLs, the cognitive-motor dual-task paradigm allows real-time assessment of task prioritization. Prioritization has been examined largely during lower-limb motor tasks (i.e., walking) while performing a cognitive task (Maclean et al., 2017; Yogev-Seligmann et al., 2012). Psychological theories of aging—such as selection, optimization, and compensation—suggest that when faced with tasks that exceed one’s available resources, individuals generally, and older adults more specifically, will prioritize the task with the higher immediate functional value (Li et al., 2001; Rapp et al., 2006). Generally, older adults and individuals with knee osteoarthritis have been found to prioritize their balance—and thus reduce attention to the cognitive task (Abdallat et al., 2020; Yogev-Seligmann et al., 2012). However, important deviations have been observed. For example, cognitive tasks may be prioritized if balance is not threatened (Schaefer, 2014). To the best of our knowledge, the dual-task paradigm has yet to be applied to upper-limb IADL performance. On the one hand, given that an upper-limb task does not challenge balance, the cognitive task may be prioritized. On the other hand, in the context of arthritis in the hands, individuals may prioritize the motor task in order to perform an upper-limb task comfortably without pain.

Although IADLs may require more attention than lower-limb motor tasks such as walking, once integrated into daily routines IADLs such as dishwashing may require few attentional resources (Bank et al., 2018). Task adaptation may be even more important in the context of living with arthritis, as experiences of pain can place constraints on daily activities. Those with arthritis have been shown to modify tasks to compensate for their limitations (Fried et al., 1996; Katz & Morris, 2007; Zhang et al., 2020). Thus, we expect that the ability to multitask while dishwashing will improve as individuals adapt to our novel standardized dishwashing protocol.

Individual differences in cognitive and physical resources, however, may differentiate not only the extent of dual-task cost (DTC; Liu-Ambrose et al., 2009), but also the prioritization of either the motor or cognitive tasks (Yogev-Seligmann et al., 2012). Given finite available resources to commit to an IADL, DTC in motor and cognitive tasks should be higher among those with fewer cognitive and physical resources, indicating difficulty in multitasking. Those with fewer physical resources may compensate by prioritizing the motor task (Gignac et al., 2002; Li et al., 2001). Meanwhile, individuals with fewer cognitive resources may inaccurately assess their physical ability and prioritize the cognitive task (Yogev-Seligmann et al., 2012).

In the current study, we examine the cognitive and motor performance components of dishwashing. Our purpose is threefold: (a) to estimate DTC of cognitive and motor performance under increased demand in an upper-limb dishwashing IADL, (b) to examine the prioritization and tradeoff of the motor and cognitive components in dual-task trials, and (c) to identify factors associated with individual differences in dual-task performance.

## Research Design and Methods

### Participants

Sixty women aged 59–91 (*M* = 67.93, *SD* = 6.77) where 97% reported diagnoses of arthritis (89% osteoarthritis; 5% rheumatoid arthritis; 6% other) and 3% reported weakness in their dominant hands were recruited from a university’s health research participant registry. Participants who regularly washed dishes by hand (53% washed dishes by hand daily), had access to email, were able to stand without an assistive device and carry a 5 lb backpack, and had no known allergies to peanut butter, latex, or cosmetic creams were included in the study. This study protocol was approved by the university’s Institutional Review Board.

### Procedure

**Figure 1.**
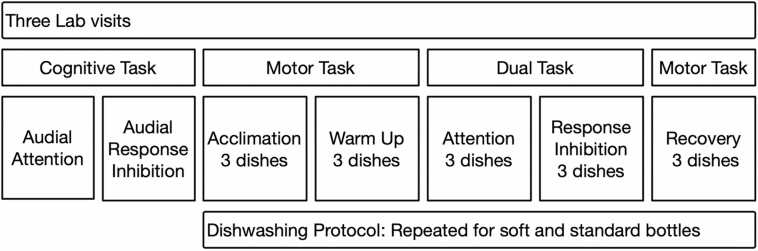
Study design. The three lab visits were separated by 1 week and designed to mimic the process of adaptation to a novel standardized dishwashing task and the soft soap dispensing package. The dishwashing protocol stages were completed twice within each lab visit. The order of bottle use was randomized across participants.

The study employed a within-person design to examine the effects of increased motor and cognitive demands during a dishwashing activity. Our study design, illustrated in Figure 1, consisted of three lab visits that were separated by 1 week. At each visit, participants initially completed two baseline cognitive tasks and then a five-stage dishwashing protocol that included three single (motor) and two dual (cognitive-motor) task stages.

The first two stages of the standardized dishwashing protocol focused on the motor task and included acclimation to the dishwashing protocol and then the warm-up stage. Two dual-task stages followed that increased cognitive demand by attention and response inhibition tasks. The final stage assessed recovery in the motor task. Three plates were washed in each stage and instructions were given at the beginning of each stage. The plates were 8 in. in diameter and weighed 13 oz. Each plate was dirtied by smearing a “Z” that spanned the diameter of the plate with one tablespoon of peanut butter. For each plate, participants were instructed to take a sponge from the researcher on their left, dispense the soap on the sponge, pick up the plate from a rack to the left of the sink, wash the plate until it was clean, place the clean plate on a rack to the right of the sink, and then hand the sponge to the research assistant. To ensure that soap was dispensed to wash each dish, a new sponge was used for each plate. Participants were instructed to clean plates to their personal standards. Participants were able to place the bottle on either side of the sink and use the hand(s) of their choice to complete the dishwashing task.

The five-stage dishwashing protocol was completed twice, within each lab visit, once using an 8 oz standard soap dispensing bottle and once using a novel *soft bottle*, designed for ease of use, of equal dimensions. Participants visited the lab on three occasions. To allow familiarization to the novel bottle, they were instructed to use the soft bottle at home between visits when washing dishes by hand (mean use = 63% of days between lab visits). At each lab visit, the amount of soap in the bottles was measured and adjusted to simulate extended bottle use, as dispensing soap is known to be more difficult in nearly empty bottles. The bottles were full during the first lab visit (~269 g), half-full at the second lab visit (126.5 g), and nearly empty at the third lab visit (56.5 g). The broader purpose of the study was to integrate behavioral and physiological dimensions of experience. Thus, electromyography, electrodermal activity, and facial expression data were also recorded, but are not reported here.

### Cognitive Demand

Cognitive demand was increased in stages three and four of the dishwashing protocol using audial attention and response inhibition tasks. During other stages, participants washed plates with pre-set segments of a popular public radio show “Car Talk” playing (68% of participants reported listening to radio, television, or podcasts while washing dishes at home). The third stage included an attention task. Participants were instructed to say “yes” every time they heard a prerecorded doorbell (target tone) that was overlaid on the audio soundtrack. The fourth stage included a go/no-go response inhibition task, which required differential response to two doorbells—the target tone that maintained the same pitch (500 Hz), and the false doorbell that changed pitch (from 500 to 300 Hz). Participants were instructed to say “yes” upon hearing the target tone, but to ignore a false doorbell. Participants’ ability to hear and distinguish the two tones was confirmed at each lab visit. To confirm that participants continued to listen to the radio segment and assess potential effects of the additional cognitive tasks, participants were asked to recall the caller’s gender, location, and the type of car trouble at the end of dual-task attention and response inhibition stages. Recall of the radio segment was also assessed in the initial baseline segments before the dishwashing protocol.

### Motor Demand

Motor demand was manipulated via differences in the ergonomic properties of the 8 oz bottles that were used to dispense soap during the dishwashing protocol. The *standard bottle* was an 8 oz bottle of dishwashing soap that is widely available in supermarkets. Video footage from the study revealed that participants dispensed soap by either squeezing or tipping the standard bottle. The *soft bottle* was made of more pliable plastic. Although it held the same amount of soap as the standard bottle, the soft bottle was lighter, conformed to the shape of the hand, and required squeezing to dispense soap, which was especially difficult when the bottle was nearly empty. The dishwashing protocol was repeated for each bottle. The order of bottle use was randomized across participants.

### Measures of Performance

Cognitive and motor task performance, as well as compensatory movements, were timed and coded using a frame-by-frame review of video and/or audio recordings.

#### DTC in motor performance

Dishwashing time—the total time to wash the three plates within each stage—measured motor performance. Timing began when participants took the sponge from the researcher’s hand at the beginning of each stage and ended when the third and final plate was placed on the rack. Participants were instructed to wash the plates until they were satisfied with their cleanliness and were not explicitly informed that their performance was timed. DTC in motor performance was calculated from the duration of the stages as follows: [(attention − warm-up)/warm-up] and [(response inhibition − warm-up)/warm-up].

#### DTC in cognitive performance

For the cognitive performance measures, we assessed the time lapse between the initiation of the target tone and the moment when the participant began to respond yes. During the go/no-go task, only response times for correct responses were analyzed. Baseline cognitive performance in the attention and response inhibition tasks was measured at each lab immediately before participants began the dishwashing activity. DTC in cognitive performance was calculated from the average response times of stages as follows: ([attention − attention baseline]/attention baseline) and ([response inhibition − response inhibition baseline]/response inhibition baseline).

#### Participant errors

Participants’ adherence to the dishwashing protocol was video-recorded to track alternative compensatory strategies to increased demand. Dishwashing errors included (a) deviation from the standardized dishwashing protocol, (b) failure to clean all peanut butter from plates, and (c) dropping/fumbling with bottle, sponge, or plate. Response time errors included incorrect recall of Car Talk content and incorrect tone responses such as failing to respond “yes” to the target tone or responding to the false tone.

### Processes of Adaptation

The three visits to the lab provided insight into participants’ adaptation to the dishwashing protocol. Participants were assumed to be naïve to the standardized dishwashing protocol and soft bottle during the first lab visit. Participants were instructed to use the soft bottle while washing dishes at home between lab visits to allow familiarization to the novel bottle. Dishwashing circumstances were assumed to be optimal during the second lab visit, as the bottle was still half-full and the soft bottle and dishwashing protocol were more familiar. The bottles were nearly empty at the third lab visit. The nearly empty bottles were expected to increase the motor demand of dispensing soap and diminish adaptation effects. Due to the soft bottle’s known property of being difficult to use when nearly empty, we expected bottle differences in DTC to be amplified at the third lab visit.

### Individual Differences in Resources

#### Physical ability

Individual differences in physical ability were characterized using two standard performance measures, one assessing upper-limb function (grip strength) and one assessing lower-limb function (timed 6-m walk). The faster gait speed of two trials (m/s) was used. Grip strength was measured on the dominant hand using a digital dynamometer (TSD121C Hand Dynamometer; BIOPAC Systems, Inc.); the average of two trials was used. Other self-reported measures of physical ability (described in Table 1) were the kitchen dimension of the Cochin Hand Function Scale (higher scores indicate greater difficulty) and a count of self-reported mobility and IADL/ADL functional limitations. Participants also reported pain while washing dishes yesterday at the beginning of each lab visit and current pain after completing each five-stage dishwashing protocol on a scale from 0 (*no pain*) to 6 (*worst possible pain*).

**Table 1.** Study Means and Interitem Correlations for Indicators of Physical and Cognitive Ability

Indicators	*M* (*SD*)	1	2	3	4	5	6	7	8	9	10
1. Age	67.93 (6.77)										
*Physical ability*											
2. Gait speed	1.21 (0.33)	−0.33									
3. Grip strength	13.09 (4.63)	−0.14	0.20								
4. Cochin	0.34 (0.43)	−0.24	−0.03	−0.51							
5. Functional limitations	3.92 (2.57)	−0.02	−0.30	−0.32	0.33						
6. Pain yesterday	1.69 (0.61)	−0.04	−0.16	−0.33	0.53	0.48					
7. Pain today	0.98 (0.81)	−0.25	−0.07	−0.42	0.57	0.59	0.79				
*Cognitive ability*											
8. Trail making A	29.01 (8.84)	0.44	−0.35	−0.25	0.03	0.09	0.01	−0.03			
9. Trail making B	62.10 (27.17)	0.38	−0.32	−0.24	0.06	0.25	0.19	0.08	0.61		
10. Word recall	12.92 (3.24)	−0.08	0.07	0.09	0.01	0.20	0.20	0.16	−0.10	0.04	
11. Word fluency	23.60 (6.79)	−0.52	0.38	0.05	0.01	−0.13	0.01	0.14	−0.40	−0.47	0.25

*Notes:* Gait speed = meters/second; grip strength = kilograms; Cochin = Cochin kitchen scale (range difficulty = 0–5); functional limitations = count of limitations (range = 0–13); pain yesterday = average across labs of self-reported recall of pain (range = 0–6); pain today = average pain reported for both bottles and all labs (range = 0–6); trail making A and B = seconds required to complete the task; word recall = *N* correctly recalled words; word fluency = *N* unique animal names within 1-min time period.

#### Cognitive ability

The trail making B test, a measure of executive function that involves connecting alternating numbers and letters in order (Tombaugh, 2004), was chosen to examine individual differences in cognitive ability. Higher scores indicate poorer performance and diminished ability. Other tests of cognitive ability included trail making A, immediate and delayed word recall, and word fluency, which are described in Table 1.

#### Missing data

All 60 participants attended the three lab visits. Out of the 360 calculations for each motor and cognitive DTC, 32 (9%) and 47 (13%) instances of DTC could not be calculated due to missing data for the motor and cognitive tasks, respectively. Missingness was due largely to video equipment failure. Participants with complete and partial data were included in the following analyses using full-information maximum likelihood.

### Statistical Analysis

DTCs were computed as described above. A total of four linear mixed-effects models, which nested observations within bottles, labs, and persons, were run: two models estimated motor DTC (change in dishwashing time during the attention and response inhibition stages relative to warm-up) and two models for cognitive DTC (change in response time during the attention and response inhibition stages relative to respective baselines). Fixed effects for bottle, lab visit, and order of bottle used were included in all analyses to estimate the effects of bottle use and adaptation to the tasks across lab visits. The standard bottle at the second lab visit was the reference condition. Planned interactions across fixed effects were tested and reported if meaningful. Significant interactions were included in the final estimates of DTC. All analyses were completed in R. Analyses with continuous outcomes were performed using the lme4 and lmerTest packages. Individual differences were explored using the emmeans package. The significance level was set to α = 0.05 for planned comparisons of DTC across bottle use and lab visits. Analyses with binary outcomes were performed using the glmer function for generalized linear mixed model fit via maximum likelihood with Laplace approximation.

## Results

Our purpose in this study was to examine (a) DTC in cognitive and motor performance, (b) the prioritization of cognitive and motor tasks, and (c) variation in DTC across individual differences in cognitive and motor resources. To address these aims, we first describe the sample to situate the results within the context of the participants’ limitations and abilities (Table 1). Compared to national norms, our sample had significantly faster trail making B scores (Tombaugh, 2004), weaker grip strength, and faster gait speed. The recruitment criteria coupled with the demands of the study resulted in the sample including women at risk of IADL limitations based on their grip strength (71%), but not gait speed (Lee et al., 2018).

**Figure 2.**
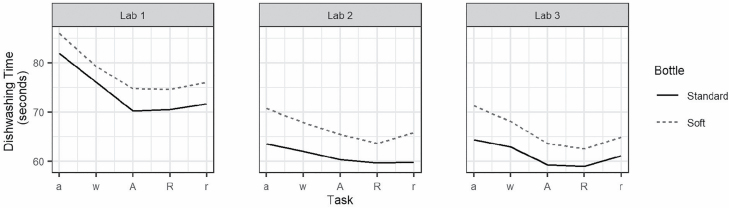
Trajectories of mean dishwashing time in seconds over the course of each lab visit, separated by bottle type. a = acclimation; w = warm-up; A = attention task; R = response inhibition task; r = recovery.

Before estimating DTC, we examined temporal trends in dishwashing within and across lab visits (Figure 2). A multilevel analysis of the five protocol stages for each of the two bottles showed that participants became faster across lab visits. Within lab visits, participants took on average nearly 5 seconds longer when using the soft bottle compared to the standard bottle (*p* < .001). For both bottles, dishwashing time showed a u-shaped trajectory across the protocol stages. Dishwashing time was slowest during the acclimation stage, sped up during warm-up, was faster still during the dual-task stages, and then slowed again during the recovery stage (warm-up vs. recovery: *p* > .05).

### DTC and Prioritization of Motor and Cognitive Tasks

**Figure 3.**
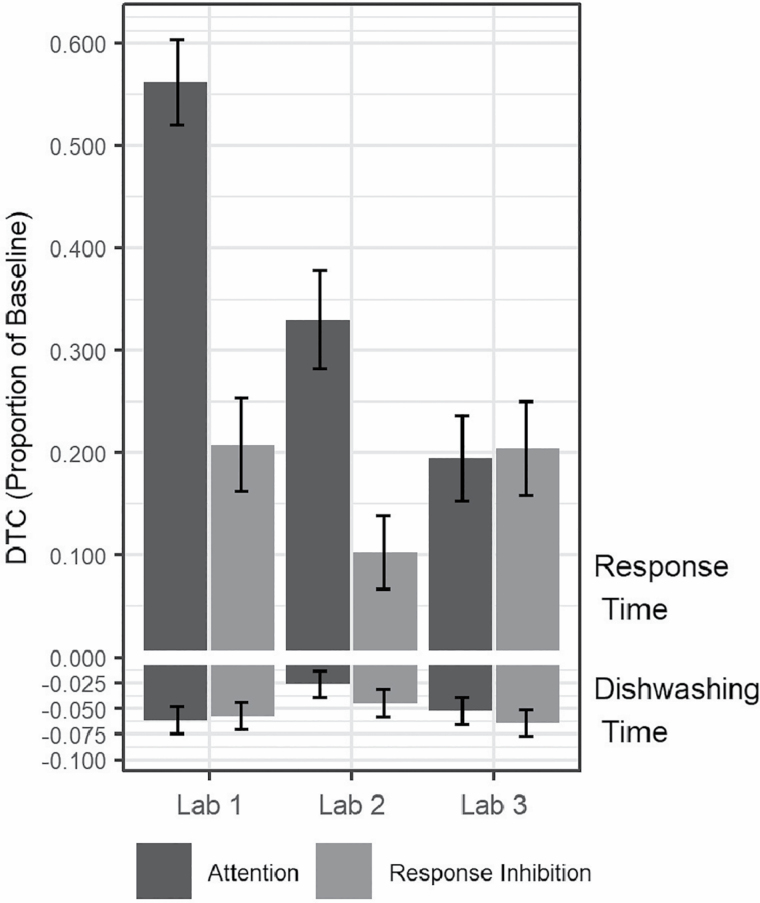
Estimated marginal means and 1 standard error bars for simultaneous dual-task cost in cognitive (response time) and motor (dishwashing time) tasks across the three lab visits and attention and response-inhibition dual-task stages. Values represent a proportional change from each task’s respective baseline. DTC = dual-task cost.

Analysis of DTC in the motor task showed that, contrary to our expectations, dishwashing time decreased during the attention stage, resulting in a negative DTC (a dual-task benefit [DTB]). This model was parameterized so that the intercept represents DTC at the second lab visit while using the standard bottle. Thus, a statistically significant intercept means that DTC for this reference condition was significantly different from zero. As shown in Figure 3, compared to the second lab visit (DTC = −0.03, *SE* = 0.01, *p* < .05), DTB was significantly more pronounced during the first (DTC = −0.06; *b* = −0.04, *SE* = 0.01, *p* < .01) and third (DTC = −0.05; *b* = −0.03, *SE* = 0.01, *p* < .05) lab visits. Dishwashing time was also faster during the response inhibition stage relative to the warm-up stage, again resulting in DTB (DTC = −0.04, *SE* = 0.01, *p* < .001). The extent of DTC in the response inhibition stage did not systematically vary across the three lab visits. Although the time required to dispense soap was longer for the soft than the standard bottles, the DTC in motor performance during the attention and response inhibition stages did not systematically vary across bottles or the two sessions within a lab visit.

Due to the complexity of the motor task, we also examined differences in the likelihood of dishwashing errors during the attention and response inhibition study stages compared to the warm-up stage. On average, out of 24 total dishwashing stages, participants failed to fully clean all three plates in 75% (*SD* = 26%), deviated from the dishwashing procedure in 10% (*SD* = 10%), and dropped or fumbled with the bottle, sponge, or plate in 3% (*SD* = 5%) of stages. Multilevel logistic regression showed that, compared to the warm-up stage, participants were more likely to deviate from the study protocol during both dual-task stages and fail to fully clean the dish during the response inhibition stage. This suggests that participants responded to the dual tasks in part by lowering their standards of a “clean dish.” The likelihood of these errors did not vary systematically across lab visits. Sensitivity analysis showed evidence of DTB to remain after adjusting for the within-person change in dishwashing errors.

Analysis of DTC in the cognitive task revealed that response time increased from the attention baseline to the attention protocol stage. Similar to our analysis of DTC in the motor task, in these models, the intercept represents DTC at the second lab visit while using the standard bottle. An adaptation effect was found for the attention task. Compared to the second lab visit (DTC = 0.33, *SE* = 0.05, *p* < .001), the extent of DTC for attention was significantly higher during the first lab visit (DTC = 0.562, *b* = 0.232, *SE* = 0.04, *p* < .001) and lower during third lab visit (DTC = 0.194, *b* = −0.136, *SE* = 0.04, *p* = .001). DTC also increased during the response inhibition protocol stage relative to response inhibition baseline (DTC = 0.13, *SE* = 0.03, *p* < .001). Differences in response inhibition DTC across lab visits were not consistent with adaptation. DTC was higher during the third lab visit (DTC = 0.21, *b* = 0.081, *SE* = 0.03, *p* < .05) than during the second lab visit but did not differ systematically between the first and second lab visits. Systematic differences in cognitive DTC were not found across bottles or across the two dishwashing sessions.

Response time errors included incorrect recall of the Car Talk segment and incorrect tone responses. On average, out of 18 cognitive task trials, participants failed to recall the Car Talk episode in 25% (*SD* = 13%) and responded to tones incorrectly in 23% (*SD* = 17%) of stages. The likelihood of both response time errors increased during the dual-task stages relative to baseline stages and decreased across lab visits. Estimates of cognitive DTC remained significant after adjusting for response errors.

Taken together, our finding that the time to wash three plates sped up while cognitive response time slowed down during the dual-task protocol stages suggests that participants prioritized the motor over the cognitive tasks. As shown in Figure 3, DTC in response time during the attention stage improved across lab visits, resulting in a diminished difference in the magnitude of DTC across the cognitive and motor tasks by the third lab visit. Evidence of prioritization remained after accounting for response errors and nonadherence to the protocol.

### Individual Differences

**Figure 4.**
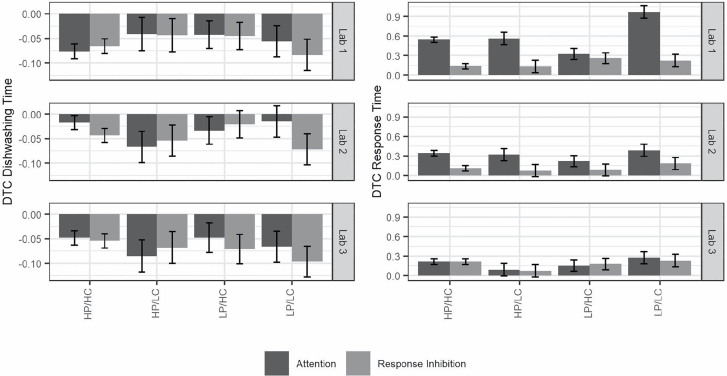
Dishwashing and response time dual-task cost across physical and cognitive ability profiles. Estimated marginal means and 1 standard error bars (*SE* computed within-group) for dual-task cost (DTC; proportional change) using all observations, grouped by ability profile, lab visit, and dual-task type. HP/HC = high physical/high cognitive, *N* = 34; HP/LC = high physical/low cognitive, *N* = 7; LP/HC = low physical/high cognitive, *N* = 9; LP/LC = low physical/low cognitive, *N* = 7.

Gait speed and standardized trail making B times were added to our models to examine the associations between DTC, prioritization, and individual differences in resources. Gait speed was not significantly related to individual differences in motor or cognitive DTC. After removing a high-influence trail making B outlier, higher trail making B scores were related to greater response time DTC (*b* = 0.26, *SE* = 0.10, *p* = .02). To explore individual differences in the combination of motor and cognitive abilities, participants were grouped by established gait speed (Lee et al., 2018) and trail making B (Tombaugh, 2004) cut-points. Group differences in DTC were identified via visual inspection of Figure 4. The main findings reported above—increased dishwashing speed at the cost of response time—were present for all groups. The DTB (negative DTC) in the motor task did not differ systematically across groups. Consistent with the expectation that DTC would be highest among those with fewer physical and cognitive resources, the largest response time DTC was observed in the low physical/low cognitive ability group during the first lab visit. For all groups, except the low cognitive/high physical ability group, response time DTC was greater for the attention than the response inhibition stage during the first lab visit. Across groups, overall response time DTC decreased during the second and third lab visits, as did the differences between attention and response inhibition DTC. Additionally, mean differences in response time DTC across groups became less pronounced during the second and third lab visits.

## Discussion and Implications

IADLs are essential to maintaining independence and are known to involve both cognitive and physical resources (Rajan et al., 2013). As age-related declines in physical and cognitive resources affect multitasking during complex tasks, prioritization becomes an important strategy to meet the demands of daily life (Rapp et al., 2006). In older adults with arthritis, the importance of navigating motor tasks comfortably is enhanced, which could have implications for prioritization and adaptation of upper-limb motor tasks. To examine the change in cognitive and motor performance and the prioritization of tasks under increased demand, we added a dual-task protocol to a standardized dishwashing task in a sample of older women with arthritis. We found cognitive performance to slow while motor performance sped up during the dual-task stages, suggesting that this sample of women with arthritis prioritized the motor over the cognitive task. We also found evidence of adaptation, as performance times for the cognitive and motor tasks decreased and evidence of prioritization was diminished by the third lab visit. Individual differences in DTC associated with cognitive and physical ability were the most pronounced during the first lab visit.

Although timed IADL performance is commonly used as an indicator of everyday cognitive function, this is the first study, to the best of our knowledge, to apply a dual-task protocol to IADL performance (Jefferson et al., 2006; Wesson et al., 2016). In contrast to the timed IADL task, dual-task protocols manipulate demand to examine within-person changes in performance as the task becomes more difficult. In contrast to previous research on timed IADL performance, dishwashing time in this study did not vary systematically across individual differences in executive function or physical ability. Furthermore, increased cognitive demand resulted in faster, rather than slower, motor performance. Although the soft bottle required more time to dispense soap than the standard bottle, DTC in motor performance did not systematically respond to change in motor demand.

We acknowledge that, in addition to the dual-task condition, performance fatigue, increases in pain, adaption, and motor demand could also influence dishwashing time. Dishwashing time became faster, rather than slower, across dishwashing stages and lab visits, which contrasts expectations for a fatigue or pain response, but aligns with dual-task response and adaptation. Additionally, although the soft bottle required more time to dispense soap, the magnitude of the speeding up effect did not systematically vary across bottles. Thus, we conclude that, in the context of this study, faster dishwashing time was in response to increased cognitive demand and was resilient to increased motor demand. The findings from this study warrant further investigation into an intraindividual change in upper-limb IADL performance to determine whether these findings generalize to populations with greater variability in cognitive ability and fewer hand arthritis limitations. Following previous research on DTC and executive function (Liu-Ambrose et al., 2009), we expect that DTC would be higher among those with compromised cognition and with physical limitations that increase the amount of attention necessary to complete the motor task.

Participants in this study prioritized the motor over the cognitive elements of the task. This finding was unexpected because older adults have been shown to prioritize the cognitive task in lower-limb activities (e.g., walking) when their balance is not threatened (Maclean et al., 2017; Schaefer, 2014; Yogev-Seligmann et al., 2012), as was the case in our upper-limb task. Dishwashing differs from walking in a population of women with arthritis in three important ways (Schaefer, 2014). First, dishwashing is an upper-limb motor task and the need to maintain postural stability is less pronounced than in a walking task (Bank et al., 2018). Second, dishwashing is likely to require more attentional resources than walking (Jefferson et al., 2006). Third, completing a novel dishwashing task with arthritis in the hand could necessitate greater attention to avoid pain (Zhang et al., 2020). It is possible that complex tasks such as washing dishes have high costs to failure. For example, a desire to complete the task without dropping the bottle or breaking the plate could also mandate prioritizing the motor task. In this study, changing the soap dispensing bottle failed to systematically affect DTC, let alone prioritization. Future research should identify new methods to increase or decrease the stakes in the context of an ecologically valid upper-limb motor task.

The prioritization of the motor task could also be specific to this local sample of women with hand arthritis. Our sample was robust in gait speed performance, due to the physical requirements of the dishwashing protocol, but had limited handgrip strength, which could increase the stakes of the motor task. The prioritization of the motor task that was observed in this study could also be specific to individuals with arthritis in the hands, where attention to navigating a task comfortably requires resources to be assigned to the motor task over the cognitive task. Consistent with this interpretation, prioritization was greatest in participants with fewer physical and cognitive resources. Taken together, although we found the motor task to be prioritized over the cognitive task, we expect that the mechanisms driving this prioritization are distinct in upper- and lower-limb activities. Our findings warrant additional research that compares upper-limb tasks across individual differences in upper-limb ability.

Adaptation to the demands of everyday life is an important strategy for individuals who live with arthritis (Fried et al., 1996; Katz & Morris, 2007). Physical pain constrains daily activities (Abdallat et al., 2020) and our standardized dishwashing protocol imposed demands on participants by mandating a specific sequence of actions while washing dishes. In this study, performance in the motor and attention tasks, but not the response inhibition task, improved across the three lab visits. Additionally, although the bottles were nearly empty and ostensibly most difficult to operate during the third lab visit, the difference in DTC across the motor and cognitive tasks—our measure of prioritization—was less pronounced. Our findings suggest that, given time to adapt, motor and cognitive attention components of IADL performance can be maintained, even in the context of increased motor demand. Additional research is needed to examine processes of adaptation in tasks that require response inhibition.

Our expectation that DTC and the need for prioritization would be greatest among those with fewer cognitive and physical resources was partially supported. Although slower IADL performance has been associated with individual differences in cognitive ability (Wesson et al., 2016), we did not find individual differences in either cognitive or physical ability to predict DTC in the motor task. However, combinations of physical and cognitive resources were important. In this study, the greatest DTC and evidence of prioritization were found among those with lower physical *and* cognitive ability. However, these differences in DTC were largely diminished by the third lab visit, suggesting that given enough time to adapt to the dishwashing protocol, those with more limitations can perform similarly to those with fewer limitations. Our findings illustrate how processes of adaptation may assist those with limited cognitive and physical ability to compensate for their limitations.

This investigation of DTCs and prioritization of motor and cognitive tasks during a standardized dishwashing activity should be viewed within the context of its limitations. First, the study recruitment criteria resulted in positive selection. It is important for future research to examine the intraindividual response to increased demand in populations with a broader range of physical and cognitive abilities. Additionally, our study examines DTC in a sample of women with arthritis while dishwashing. It is possible that women would dedicate more attention to a gendered task such as dishwashing than men would. Future research should investigate the tradeoff between motor and cognitive resources in ecologically valid contexts. We also acknowledge that allowing participants to take home and use the soft bottle between lab visits may have introduced additional practice effects. Follow-up analyses of measured change in bottle volume following the at-home practice periods showed a change in bottle volume to decrease, rather than remain stable or increase, across the two practice periods. This suggests that improvement in DTC across lab visits was driven more by processes of adaption than by practice. Finally, although differences in DTC in the motor and cognitive tasks were clear in this study—where dishwashing time decreased as response time increased, there are also limitations in our ability to directly compare DTC in the motor and cognitive tasks. DTC in the cognitive and motor tasks was scaled as proportional change, but because the motor task took longer than the cognitive response task, the differences in the magnitude of this proportional change are still not directly comparable.

In conclusion, we found older women with arthritis to prioritize the motor task of washing dishes over the completion of a cognitive task. Processes of adaptation allowed for accommodations that improved performance, reduced the need for prioritization, and allowed for compensation of diminished physical and cognitive abilities. This study of cognitive and motor response to increased demand during an upper-limb IADL illustrates the value of examining multiple measures across different phases of a task. Although the required analytic and theoretical framework is more complex, this study highlights the potential to gain a richer understanding of how multiple factors simultaneously affect IADL performance.
