# Corrigendum to: Assessment of Cognitive-Motor Performance Costs, Task Prioritization, and Adaptation to Dishwashing Under Increased Demand in Older Women With Arthritis

**DOI:** 10.1093/geroni/igab005

**Published:** 2021-02-10

**Authors:** Shannon T Mejía, Karen E Nielsen, Vineet Raichur, Alicia G Carmichael, Eugene Tavares, Jennie Jarvis, Jacqui Smith, Rich Gonzalez

**Affiliations:** 1 Department of Kinesiology and Community Health, University of Illinois at Urbana-Champaign, Champaign, Illinois, US; 2 Department of Population Health Sciences, School of Public Health, Georgia State University, Atlanta, Georgia, US; 3 Institute for Social Research, University of Michigan, Ann Arbor, Michigan, US; 4 Procter & Gamble, Cincinnati, Ohio, US

In the original research article, “Assessment of Cognitive-Motor Performance Costs, Task Prioritization, and Adaptation to Dishwashing Under Increased Demand in Older Women With Arthritis,” DOI: 10.1093/geroni/igaa059, co-author Eugene Tavares’ name and academic degree were misspelled as “Tavare” and “BSs”, respectively. It has since been corrected.

